# Total Water and Energy Intake Among Preschool Children in China: A Cross-Sectional Analysis Based on National Survey Data

**DOI:** 10.3390/nu17162645

**Published:** 2025-08-15

**Authors:** Zhencheng Xie, Wanyi Yang, Lishan Ouyang, Minghan Fu, Hongliang Luo, Yitong Li, Ye Ding, Zhixu Wang

**Affiliations:** 1 Department of Maternal, Child and Adolescent Health, School of Public Health, Nanjing Medical University, Nanjing 211166, China; zhenchengxie@njmu.edu.cn (Z.X.); yangwanyi@stu.njmu.edu.cn (W.Y.); ouyanglishan@stu.njmu.edu.cn (L.O.); fuminghan@stu.njmu.edu.cn (M.F.); 2 Danone Open Science Research Center for Life-Transforming Nutrition, Shanghai 201204, China; hongliang.luo@danone.com (H.L.); yitong.li@danone.com (Y.L.); 3 The Institute of Nutrition and Food Science, Nanjing Medical University, Nanjing 211166, China

**Keywords:** Chinese preschool children, water intake, energy intake, adequate intake

## Abstract

**Background:** Adequate hydration for preschool children (36–72 months) is critical for their healthy growth, cognitive development, and long-term well-being. However, there is still a lack of reliable baseline data in China to inform water intake guidelines for this age group. **Methods:** In this study, a cross-sectional analysis was conducted using data from the 2018–2019 Dietary Survey of Infants and Young Children in China, including 676 healthy preschool children. Water and energy intake were estimated using four-day food diaries. Their daily total water intake (TWI) and total energy intake (TEI) were evaluated, and the contributions of beverages and foods to TWI and TEI were analyzed, respectively. The TWI was compared with the adequate intake (AI) set by the Chinese Nutrition Society, and the correlations between water and energy intake were explored. **Results:** The results show that the median daily TWI was 1218 mL, with 667 mL (55.7%, r = 0.824) from beverages and 520 mL (44.3%, r = 0.691) from foods. Among beverages, plain water (74.4%, r = 0.903) and milk and milk derivatives (MMDs, 20.9%, r = 0.443) were the main contributors, while staple foods, dishes, and soup contributed the majority of the water from foods. Only 19.4% of children’s TWI met the AI level, and their water and energy intake was significantly higher than those who did not. The median daily TEI was 994 kcal, with 739 kcal (77.2%, r = 0.806) from foods and 198 kcal (22.8%, r = 0.644) from beverages. MMDs accounted for 83.1% of beverage energy (r = 0.880). Boys consumed more beverages than girls, especially in the 37–48 months group. **Conclusions:** As the first nationally representative study of TWI among Chinese preschool children, these findings reveal a substantial gap between actual intake and current recommendations, and highlight the need to revise reference values and improve hydration guidance in early childhood.

## 1. Introduction

Water is the most abundant liquid in nature and serves as the primary component of all beverages and most foods. Despite being a fundamental nutrient essential for life, water is often overlooked in dietary recommendations and educational resources. Research indicates that even mild dehydration can negatively affect cognitive function and mood in preschool children [1]. Furthermore, it may impair brain structure and function in adolescents [2]. Severe dehydration may lead to reversible acute kidney conditions, such as tubular ischemia [3]. Due to their larger body surface area, higher activity levels, and rapid growth, children have a greater need for water and are more susceptible to dehydration compared to adults [4].

Healthy hydration has become a global public health priority, with increasing emphasis on plain water consumption and limiting sugar-sweetened beverages (SSBs) [5,6]. Nevertheless, many children fail to meet the recommended water intake. A review covering 19 countries found wide disparities in water intake adequacy, with national noncompliance rates among children aged 4–13 years ranging from 10% to 98%, and an overall rate of approximately 60% [4]. These differences may be influenced by various factors, including climate, culture, water safety, and the availability and affordability of beverages and foods. Additionally, local policies and the methods used in surveys can also contribute to these differences. This suggests that total water intake (TWI) among children varies by country, and the contributions of beverages and foods to TWI can differ significantly as well.

The preschool period is a critical time for fostering healthy dietary habits. The establishment of healthy and sustainable drinking habits, maintenance of adequate fluid intake, and optimal hydration status are of paramount importance for children [4]. Baseline data on water intake are essential for formulating recommendations on adequate intake (AI) levels. Therefore, understanding the TWI and its sources of preschool children is of great value. However, there is a serious lack of baseline data on water intake among this age group in China, which poses challenges for developing scientifically water intake recommendations. The Chinese Nutrition Society (CNS) estimates that the appropriate TWI for preschool children is 1600 mL/day. This estimate is based on the average adult water intake (1700 mL/day), considering the weight ratio between children and adults as well as growth coefficients for children [7]. However, many researchers express concerns about the applicability of these recommendations since they are not grounded in actual survey data.

To address this evidence gap and provide population-specific data for national guidelines, we conducted this study. This study utilized data from the 2018–2019 cross-sectional Dietary Survey of Infants and Young Children in China (DSIYC). Specifically, the primary objective was to assess daily TWI and total energy intake (TEI), as well as the contributions of various beverages and foods to both. In addition, we sought to evaluate the adequacy of TWI by comparing the observed values with the age-specific AI recommended by the CNS. Finally, we explored the associations between water and energy intake from different dietary sources.

## 2. Materials and Methods

### 2.1. Study Participants

The data for this study were derived from the DSIYC, collected between September 2018 and February 2019. Participants were healthy, full-term singleton children aged 36–72 months, with no diagnosed chronic diseases or congenital conditions, recruited from 11 provinces and 2 municipalities across mainland China. The study protocol was approved by the Ethics Committee of Nanjing Medical University (approval number: 2018-1123). The DSIYC employed a stratified sampling design that accounted for geographic location, local economic development, and annual birth rates. Detailed inclusion and exclusion criteria have been described in a previously published study [8]. The sample size was estimated using the following formula: $N\;=\;\frac{{\left(Z_{\alpha }\times S\right)}^{2}}{d^{2}}$. The confidence level (1-α) was set at 95%, corresponding to a *Z*_α_ value of 1.96. The standard deviation (*S*) of the daily intake of plain water for children, derived from a previous study involving children aged 4–9 from 13 countries, was 535 mL. The maximum measurement error (*d*) was set at 50 mL [9]. Based on these parameters, the formula indicated that a minimum of 440 participants was required. In our study, data from 676 children were ultimately analyzed, ensuring an accurate representation of the population. In pursuit of the purpose of this study, our analysis specifically focused on the data related to water and energy intake from various types of beverages and foods recorded during the dietary survey.

### 2.2. Assessment of Water and Dietary Intake

As previously described [8], a four-day dietary record was collected using an online diary supplemented by a validated food atlas [10], with standardized instructions provided to caregivers beforehand and trained investigators available for support during the recording process. Total fluid intake (TFI) referred to the volume of beverages consumed, whereas TWI included water from both beverages and foods. Beverages were categorized as plain water, milk and milk derivatives (MMDs), fruit and vegetables drinks (FVDs), SSBs, botanical protein drinks (BPDs), and hot beverages. SSBs included carbonated beverages, fruit- and tea-flavored drinks, milk-flavored beverages, functional beverages, and milk tea products. Food sources primarily included staple foods, dishes, porridge, soup, and snacks.

### 2.3. Statistical Analysis

Statistical analyses were performed using SPSS version 26.0 (IBM, New York, NY, USA). Continuous variables were tested for normality; those with normal distribution were reported as mean ± standard deviation, while non-normally distributed variables were summarized as median (P25–P75). Categorical variables were expressed as counts (n) and percentages (%). Group comparisons by age were conducted using the Kruskal–Wallis H test or one-way ANOVA, and by sex using the Mann–Whitney U test or independent samples *t*-test. Partial correlation analysis, adjusting for age (in months), sex, height, and weight, was applied to assess the associations between TWI, TEI, and intake from different beverage and food sources. A two-tailed *p*-value < 0.05 was considered statistically significant.

## 3. Results

### 3.1. Characteristic of the Study Participants

The mean age of the preschool children included in this study was 54.6 months, with an average height of 106.8 cm and an average weight of 18.1 kg. Participants were categorized into three age groups: 37–48 months (*n* = 224), 49–60 months (*n* = 226), and 61–72 months (*n* = 226). Significant differences were observed in age, height, and weight across the three groups (*p* < 0.05). Detailed characteristics are presented in Supplementary Table S1.

### 3.2. Daily TWI and Source Contributions

Across the study participants, the median daily TWI was 1218 mL, comprising 667 mL (55.7%) from beverages and 520 mL (44.3%) from foods. Among beverage sources, plain water was the primary contributor, accounting for 74.4% of beverage-derived water, with a median intake of 500 mL. MMDs contributed 20.9%, followed by SSBs, BPDs, and FVDs. Regarding water intake from food sources, staple foods were the largest contributors (29.4%, median 137 mL), followed by dishes, porridge, soup, and snacks.

As shown in Table 1, no significant age-related differences were observed in TWI, TFI, or water intake from most beverage and food categories, including plain water, BPDs, hot beverages, dishes, and snacks (*p* > 0.05). Except for hot beverages, soup, and snacks, the proportional contributions of beverage- and food-derived water to TWI varied significantly with age (*p* < 0.05).

As shown in Table 2, boys had significantly higher water intake from beverages and staple foods than girls (*p* < 0.05). However, no significant sex differences were observed in overall TWI, TFI, or in water intake and percentage contribution from other beverage and food categories (*p* > 0.05). Further stratification by age and sex revealed that boys aged 37–48 months had significantly higher TWI, TFI, and water intake from beverages, including plain water and MMDs, than girls in the same age group (*p* < 0.05). However, for children aged 49–60 months and 61–72 months, no significant sex differences were observed in TWI, TFI, or in water intake and distribution across beverage and food sources (*p* > 0.05). Detailed results are provided in Supplementary Table S2.

### 3.3. Comparison of TWI and Source Contributions According to Whether the AI Recommended by the CNS Was Met

According to Table 3, children were classified based on whether their daily TWI met the AI level recommended by the CNS. Group 1 (*n* = 131), comprising children who met the AI, had a median daily TWI of 1803 mL. In this group, beverage-derived water contributed 1074 mL (57.8%), exceeding the contribution from food-derived water (745 mL, 42.2%). Conversely, Group 2 (*n* = 545), whose TWI fell below the AI, had a significantly lower median TWI of 1112 mL. However, the relative proportions of beverage- and food-derived water (55.2% and 44.8%, respectively) were comparable between the two groups.

Further analysis revealed that Group 1 had significantly higher intake of plain water and MMDs compared to Group 2 (*p* < 0.05). The median values were 800 mL vs. 480 mL for plain water, and 200 mL vs. 118 mL for MMDs. Water intake from staple foods, dishes, porridge, and snacks was likewise significantly higher in Group 1 (*p* < 0.05 for all comparisons). In terms of proportional contributions, beverages accounted for a significantly greater share of TWI in Group 1. In contrast, the proportion of water derived from foods and the share of staple foods in food-based water were both significantly lower in Group 1 than in Group 2 (*p* < 0.05).

### 3.4. Daily TEI and Source Contributions

The median daily TEI among the study population was 994 kcal, with beverages contributing 198 kcal (22.8%) and foods contributing 739 kcal (77.2%). Among beverage sources, MMDs were the primary contributors, accounting for 83.1% of beverage-derived energy, with a median intake of 155 kcal. SSBs contributed 9.6%, followed by BPDs, FVDs, and hot beverages. Regarding energy intake from food sources, staple foods were the largest contributors (40.8%, 293 kcal), followed by dishes, snacks, and porridge.

As shown in Table 4, significant age-related differences were observed in TEI, energy from foods, and energy intake from MMDs, FVDs, SSBs, staple foods, dishes, and porridge (*p* < 0.05). Additionally, the proportional contributions of energy from MMDs and SSBs to beverage-derived energy varied significantly with age (*p* < 0.05).

As shown in Table 5, no significant sex differences were observed in TEI or energy intake from different types of beverages and foods (*p* > 0.05). However, the proportion of energy from snacks was significantly higher in girls (17.6%) than in boys (15.6%) (*p* < 0.05). Further stratification by age and sex indicated that among children aged 37–48 months, boys had significantly higher energy intake from beverages and MMDs, as well as a higher percentage of energy from beverages relative to TEI compared to girls of the same age (*p* < 0.05). Among children aged 49–60 months, energy intake from staple foods was significantly higher in boys than in girls (*p* < 0.05). In contrast, among those aged 61–72 months, boys had significantly lower energy intake from MMDs and a lower proportion of energy from snacks relative to energy from foods than girls (*p* < 0.05). Detailed results are provided in Supplementary Table S3.

### 3.5. Comparison of TWI and Source Contributions According to Whether the Recommended TWI Was Met

As in Section 3.3, children were classified based on whether their TWI met the AI recommended by the CNS. Group 1 had a median daily TEI of 1246 kcal, with 967 kcal (76.8%) derived from foods and 245 kcal (23.2%) from beverages. In contrast, Group 2 had a significantly lower median TEI of 919 kcal, though the proportions of energy from foods (77.2%) and beverages (22.8%) were comparable to those in Group 1.

Further analysis indicated that energy intake from MMDs, FVDs, and SSBs was significantly higher in Group 1 than in Group 2 (*p* < 0.05). Similarly, energy intake from staple foods, dishes, porridge, and snacks was significantly higher in Group 1 (*p* < 0.05 for all comparisons). In terms of proportional contributions, both the share of BPDs in beverage-derived energy and the contribution of staple foods to food-derived energy were significantly lower in Group 1 than in Group 2 (*p* < 0.05). In contrast, the contribution of energy from porridge to food-derived energy was significantly higher in Group 1 than in Group 2 (*p* < 0.05). Detailed results are presented in Table 6.

### 3.6. Partial Correlation Analysis Between Water and Energy Intake

After adjusting for age in months, sex, height, and weight, correlations between water and energy intake from various sources were assessed (Table 7). As expected, TWI showed strong correlations with TFI and water from beverages (r = 0.817 and 0.824, respectively), as well as moderate correlations with plain water and water from foods. Notably, water from beverages was highly correlated with plain water (r = 0.903), indicating that plain water constituted the major component of beverage-derived water. Meanwhile, water from foods demonstrated moderate correlations with water from dishes, soup, and snacks.

With respect to energy intake, TEI exhibited a strong correlation with energy derived from foods (r = 0.806), and moderate correlations with energy from beverages, MMDs, staple foods, dishes, and snacks. Energy from beverages was strongly correlated with energy from MMDs (r = 0.880), whereas energy from foods showed moderate correlations with energy from staple foods, dishes, and snacks.

Regarding the relationship between energy and water intake, TEI was moderately correlated with water from foods (r = 0.557), particularly with water from dishes (r = 0.531). Similarly, energy from foods was moderately associated with water from foods (r = 0.717), including water derived from dishes (r = 0.513).

## 4. Discussion

This pioneering study analyzed TWI among Chinese preschool children using nationally representative data. Specifically, it examined both the quantity and sources of TWI in children aged 36–72 months. The median daily TWI was 1218 mL, with beverages contributing 667 mL (55.7%). Among these, plain water (74.4%) and MMDs (20.9%) were the dominant sources, suggesting a healthier beverage consumption pattern compared to other populations with a higher intake of SSDs.

Numerous studies documented substantial cross-country differences in water intake among children. For instance, the average daily TWI for children aged 4–8 years was 1233 mL in France [11], 1279 mL in the United Kingdom [12], 1427 mL in Mexico [13], 1447 mL in the United States [14], and 1601 mL in Lebanon [15]. However, these findings are not fully comparable due to differences in survey methodologies, age categorizations, and measurement approaches. For instance, some studies reported only beverage-derived water intake, excluding contributions from food. In a study involving preschoolers aged 3–6 years across six European countries (Belgium, Bulgaria, Germany, Greece, Poland, and Spain), the average TFI was 1051 mL [16]. By contrast, a separate study from Spain reported a notably higher intake of 1571 mL [17]. In Latin America, children aged 4–9 years in Argentina consumed an average of 1807 mL [18], substantially exceeding intakes in Brazil (1414 mL) [18] and Mexico (1232 mL) [18]. Such discrepancies may be attributed to a range of contextual factors, including dietary culture, climate, ethnic background, beverage availability, and perceptions of water safety.

Traditional Chinese diets typically emphasize cooking methods such as steaming, boiling, and stewing, within a predominantly plant-based dietary structure. These practices result in frequent consumption of high-moisture foods, including soups and porridges. Consequently, a substantial proportion of water intake is derived from food. In this study, food accounted for 44.3% of children’s TWI, with staple foods contributing the most (29.4%), followed by soups and porridges. By contrast, Western diets typically rely on dry heat cooking methods such as baking and grilling, feature a higher proportion of animal-based foods, and include fewer liquid or semi-liquid dishes, all of which contribute to a lower percentage of water obtained from food. Numerous studies also showed marked cross-national differences in food-derived water intake: 28.5% in Lebanese children [15], 29.8% in the United States [14], 33% in the United Kingdom [19], 35% in France [19], and 35.4% in Mexico [13]. The relatively higher proportion observed among Chinese children underscores the hydration value of traditional dietary patterns. Therefore, evaluations of water intake in this population must consider contributions from moisture-rich foods, such as dishes, soups, and porridges, to avoid underestimating true hydration status.

Notably, in this study, 81.6% of children did not meet the TWI recommendation of 1600 mL/day set by the CNS, indicating that inadequate water intake is widespread in this population. This observation is consistent with findings from other studies. For example, among children aged 4–8 years, 84% in the United Kingdom [12] failed to meet the European Food Safety Authority guidelines, while the noncompliance rate was even higher in France (89%) [11]. In the United States [14], 75% of children did not reach the TWI standards set by the National Academies of Sciences, Engineering, and Medicine (formerly IOM), and the noncompliance rate based on the same criteria in Lebanon [15] also reached 74%. In Latin American countries [18], the proportions of 4–9-year-old children not meeting IOM recommendations were 38% in Mexico, 47% in Brazil, and 69% in Argentina. Collectively, these data suggest that suboptimal water intake among preschool children is a widespread global public health concern.

Gender disparities appear to further compound this widespread inadequacy in water intake. Numerous studies have consistently shown that girls are less likely than boys to meet recommended intake levels—for example, 90.3% vs. 58.5% in the Lebanon [15] and 87% vs. 66% in the United States [20]. This trend was also observed in our dataset and in other regions. The disparity is evident not only in compliance rates, but also in actual intake levels. For instance, boys aged 4–8 years had higher average daily TWI than girls in France (1280 mL vs. 1180 mL) [11], the United Kingdom (1280 mL vs. 1276 mL) [12], Mexico (1467 mL vs. 1389 mL) [13], and the United States (1477 mL vs. 1415 mL) [14]. The pattern persisted even when only fluid intake was considered—for example, in Mexico (1301 mL vs. 1169 mL) [18], Argentina (1843 mL vs. 1737 mL) [18], China (891 mL vs. 784 mL) [21], and six European countries [16], where boys consumed 1055 mL compared to girls’ 986 mL. Brazil was one of the few exceptions, with boys reporting slightly lower TWI than girls (1393 mL vs. 1432 mL) [18], potentially reflecting sociocultural or dietary factors. In our sample, boys aged 37–48 months had significantly higher TWI, TFI, and beverage-derived water intake, especially plain water and MMDs, compared to girls. Boys also consumed more dairy overall, contributing to elevated water and energy intake from these beverages. These findings underscore gender-based differences in beverage preferences and nutrient intake, potentially originating from higher physical activity levels, faster growth, and increased hydration needs among boys in early childhood. However, as children grow older, their dietary behaviors are increasingly shaped by family routines and childcare settings, leading to a reduction in gender-based disparities. These findings highlight the importance of the early preschool years as a critical window for implementing hydration interventions, emphasizing the necessity for age- and gender-specific strategies.

Regarding energy intake, this study found that MMDs were the predominant source of beverage-derived energy, accounting for 83.1%. This underscores their nutritional significance in preschoolers’ diets. Conversely, SSBs, though consumed in modest amounts, still contributed 9.6% of beverage energy and showed an upward trend with age. Some studies reported that SSBs can account for up to 8.5% of TEI in children in the United States [14], with even higher proportions documented in Mexico [13]. These beverages are closely linked to adverse health outcomes, including obesity, dental caries, and metabolic disorders [22]. Although the current consumption levels of SSBs among Chinese children remain relatively moderate, their upward trend indicates the necessity for timely public health interventions. Preventive strategies, such as caregiver education, the implementation of beverage policies in childcare settings, and limiting the accessibility of SSBs, are strongly recommended.

This study also identified a moderate correlation between total water and energy intake (r = 0.483), with particularly strong associations observed for staple foods (r = 0.723) and dishes (r = 0.682), suggesting that these traditional Chinese foods are major contributors of both water and energy. MMDs similarly served a dual function, contributing meaningfully to both hydration and caloric intake, thereby reinforcing their central role in Chinese young children’s beverage patterns. However, with age, the intake of beverages with lower nutrient density, such as SSBs and BPDs, tends to increase, potentially displacing dairy consumption and undermining both hydration and overall diet quality. Drawing from global experience, early education on healthy beverage choices, and structured guidance on fluid intake should be strengthened to promote optimal nutrition and hydration for Chinese children in their early stages.

Despite the strengths of this study, including a large sample size, multi-day dietary records, and the use of a food atlas to enhance data accuracy and representativeness, several limitations should be acknowledged. First, its cross-sectional design limits causal inference and prevents the observation of longitudinal changes in hydration behaviors as children grow older. Moreover, due to limited subgroup sample sizes, stratified analyses by region or season were not conducted, which may affect the generalizability of findings across China’s diverse settings. Future studies with longitudinal follow-up are needed to explore how early hydration patterns relate to health outcomes over time. Second, although standardized tools and training were used, caregiver-reported food records may still be subject to estimation bias, especially in quantifying liquid-based foods such as soups and porridges, as well as snacks consumed between meals or outside the home. In addition, under-reporting of total energy intake, which is commonly seen in dietary surveys, may have led to a parallel underestimation of water intake and potentially resulted in an overestimation of the prevalence of insufficient hydration. Third, the absence of objective hydration biomarkers (e.g., urine specific gravity or osmolality) limits the ability to accurately assess children’s hydration status.

## 5. Conclusions

In summary, this study provides pioneering nationally representative baseline data on water intake among Chinese preschool children. The findings reveal widespread suboptimal TWI, diverse sources of water intake, and notable age- and gender-based disparities, all of which carry important public health implications. These results offer localized evidence to inform future revisions of the Chinese Dietary Reference Intakes. Moving forward, it is imperative to improve the drinking environment via family education, policy initiatives, and standardized childcare practices to promote healthy hydration behaviors during this critical developmental stage and to bolster the long-term health of children.

## Figures and Tables

**Table 1:** Water intake (mL/day) and source-specific contributions (%) among Chinese preschool children in 2018 stratified by age group (months).

Variables	Total (*n* = 676)		37–48 Months (*n* = 224)		49–60 Months (*n* = 226)		61–72 Months (*n* = 226)	
	Median (P25–P75)	%	Median (P25–P75)	%	Median (P25–P75)	%	Median (P25–P75)	%
Total water intake	1218 (977–1530)	–	1169 (944–1053)	–	1257 (1013–1503)	–	1221 (971–1568)	–
Total fluid intake	700 (525–938)	–	719 (524–938)	–	688 (539–960)	–	691 (510–925)	–
Water from beverages	667 (500–902)	55.7 #	685 (498–908)	58.0	641 (500–894)	54.1	667 (495–911)	55.2
Plain water	500 (303–700)	74.4 #	500 (300–600)	72.4	500 (328–763)	77.3	500 (300–619)	73.5
MMDs	135 (45–219) *	20.9 #	167 (88–239)	24.4	111 (8–208)	18.3	132 (56–214)	20.1
FVDs	0 (0–0) *	0.7 #	0 (0–0)	0.3	0 (0–0)	0.6	0 (0–0)	1.0
SSBs	0 (0–24) *	2.6 #	0 (0–0)	1.7	0 (0–24)	2.4	0 (0–34)	3.5
BPDs	0 (0–0)	1.5 #	0 (0–0)	1.2	0 (0–0)	1.3	0 (0–20)	1.9
Hot beverages	0 (0–0)	0	0 (0–0)	0	0 (0–0)	0	0 (0–0)	0
Water from foods	520 (386–679) *	44.3 #	465 (363–621)	42.0	549 (425–693)	45.9	529 (382–717)	44.9
Staple foods	137 (102–187) *	29.4 #	126 (93–176)	29.5	136 (101–184)	27.3	148 (114–200)	31.5
Dishes	134 (85–200)	27.2 #	130 (81–193)	28.0	132 (81–200)	25.4	142 (88–209)	28.1
Porridge	55 (16–137) *	16.5 #	48 (18–114)	15.5	96 (32–178)	21.5	35 (8–92)	12.3
Soup	63 (25–125) *	14.7	50 (23–100)	13.8	70 (27–135)	14.6	75 (25–138)	15.8
Snacks	55 (28–92)	12.2	51 (30–92)	13.1	58 (26–89)	11.3	54 (28–99)	12.2

* Group differences in water intake from specific sources were statistically significant (*p* < 0.05); # proportional contributions of each source to total, beverage-, and food-derived water differed significantly across groups (*p* < 0.05).

**Table 2:** Water intake (mL/day) and source-specific contributions (%) stratified by sex.

Variables	Boy (*n* = 340, 50.3%)		Girl (*n* = 336, 49.7%)	
	Median (P25–P75)	%	Median (P25–P75)	%
Total water intake	1276 (1003–1559)	–	1156 (955–1492)	–
Total fluid intake	744 (528–975)	–	650 (523–888)	–
Water from beverages	703 (500–934) *	56.5	612 (500–844)	55.0
Plain water	500 (335–800)	73.9	500 (300–600)	74.9
MMDs	143 (45–225)	21.3	132 (51–208)	20.5
FVDs	0 (0–0)	0.7	0 (0–0)	0.7
SSBs	0 (0–24)	2.6	0 (0–24)	2.5
BPDs	0 (0–3)	1.6	0 (0–0)	1.4
Hot beverages	0 (0–0)	0.0	0 (0–0)	0.0
Water from foods	519 (386–668)	43.5	521 (385–685)	45.0
Staple foods	141 (107–195) *	30.4	134 (99–177)	28.5
Dishes	138 (87–204)	27.7	130 (81–199)	26.7
Porridge	52 (16–132)	15.9	57 (16–138)	17.0
Soup	63 (25–125)	14.2	65 (25–127)	15.2
Snacks	53 (27–89)	11.8	58 (31–98)	12.5

* Group differences in water intake from specific sources were statistically significant (*p* < 0.05).

**Table 3:** Water intake (mL/day) and source-specific contributions (%) stratified by whether total water intake met the adequate intake set by the Chinese Nutrition Society.

Variables	Group 1 (*n* = 131, 19.4%)		Group 2 (*n* = 545, 80.6%)	
	Median (P25–P75)	%	Median (P25–P75)	%
Total water intake	1803 (1670–1961) *	–	1112 (930–1335)	–
Total fluid intake	1115 (938–1275) *	–	635 (500–800)	–
Water from beverages	1074 (903–1238) *	57.8 #	600 (473–758)	55.2
Plain water	800 (600–1000) *	75.3	480 (300–500)	74.2
MMDs	200 (122–281) *	20.1	118 (40–200)	21.1
FVDs	0 (0–0) *	0.9	0 (0–0)	0.6
SSBs	0 (0–36) *	2.6	0 (0–24)	2.5
BPDs	0 (0–2)	1.1	0 (0–0)	1.6
Hot beverages	0 (0–0)	0.0	0 (0–0)	0.0
Water from foods	745 (614–944) *	42.2 #	475 (368–596)	44.8
Staple foods	165 (114–219) *	23.7 #	134 (98–178)	30.8
Dishes	224 (163–287) *	28.7	122 (79–173)	26.8
Porridge	90 (28–232) *	18.1	50 (16–122)	16.1
Soup	103 (60–198) *	16.5 #	53 (25–116)	14.3
Snacks	90 (51–127) *	13.0	50 (24–80)	12.0

Group 1: children whose daily TWI met the AI recommended by the CNS; Group 2: children whose daily TWI did not meet the AI. * Group differences in water intake from specific sources were statistically significant (*p* < 0.05); # proportional contributions of each source to total, beverage-, and food-derived water differed significantly across groups (*p* < 0.05).

**Table 4:** Energy intake (kcal/day) and source-specific contributions (%) stratified by age group (months).

Variables	Total (*n* = 676)		37–48 Months (*n* = 224)		49–60 Months (*n* = 226)		61–72 Months (*n* = 226)	
	Median (P25–P75)	%	Median (P25–P75)	%	Median (P25–P75)	%	Median (P25–P75)	%
Total energy intake	994 (766–1257) *	–	911 (726–1176)	–	1001 (774–1236)	–	1074 (832–1336)	–
Energy from beverages	198 (98–356)	22.8	212 (110–338)	24.2	189 (99–354)	22.5	200 (78–373)	21.7
Plain water	0 (0–0)	0	0 (0–0)	0	0 (0–0)	0	0 (0–0)	0
MMDs	155 (68–298) *	83.1 #	190 (90–331)	89.6	149 (74–268)	83.0	134 (53–264)	76.9
FVDs	0 (0–0) *	3.0	0 (0–0)	1.8	0 (0–0)	3.4	0 (0–0)	3.7
SSBs	0 (0–5) *	9.6 #	0 (0–0)	5.1	0 (0–5)	9.6	0 (0–10)	14.0
BPDs	0 (0–0)	4.1	0 (0–0)	3.4	0 (0–0)	3.7	0 (0–7)	5.3
Hot beverages	0 (0–0)	0.2	0 (0–0)	0.1	0 (0–0)	0.3	0 (0–0)	0.2
Energy from foods	739 (577–943) *	77.2	674 (499–888)	75.8	742 (587–927)	77.4	832 (625–1020)	78.3
Staple foods	293 (207–405) *	40.8	269 (184–388)	40.5	292 (207–381)	39.6	324 (241–473)	42.1
Dishes	266 (206–352) *	38.7	237 (179–317)	38.0	279 (221–361)	39.9	284 (219–386)	38.3
Porridge	6 (1–35) *	3.9	5 (1–31)	4.2	12 (3–43)	4.3	3 (1–34)	3.3
Soup	0 (0–0)	0	0 (0–0)	0	0 (0–0)	0	0 (0–0)	0
Snacks	105 (59–183)	16.6	104 (62–179)	17.4	101 (56–172)	16.2	110 (57–195)	16.2

* Group differences in energy intake from specific sources were statistically significant (*p* < 0.05); # proportional contributions of each source to total, beverage-, and food-derived energy differed significantly across groups (*p* < 0.05).

**Table 5:** Energy intake (kcal/day) and source-specific contributions (%) stratified by sex.

Variables	Boy (*n* = 340)		Girl (*n* = 336)	
	Median (P25–P75)	%	Median (P25–P75)	%
Total energy intake	1008 (762–1236)	–	963 (764–1244)	–
Energy from beverages	205 (100–358)	22.9	192 (91–356)	22.6
Plain water	0 (0–0)	0.0	0 (0–0)	0.0
MMDs	157 (70–289)	81.9	152 (68–305)	84.4
FVDs	0 (0–0)	3.1	0 (0–0)	2.9
SSBs	0 (0–5)	10.4	0 (0–5)	8.8
BPDs	0 (0–1)	4.5	0 (0–0)	3.7
Hot beverages	0 (0–0)	0.2	0 (0–0)	0.2
Energy from foods	745 (569–954)	77.1	733 (577–935)	77.4
Staple foods	299 (206–428)	41.5	287 (207–377)	40.0
Dishes	274 (207–352)	39.2	257 (204–350)	38.2
Porridge	7 (1–33)	3.7	6 (1–39)	4.2
Soup	0 (0–0)	0.0	0 (0–0)	0.0
Snacks	99 (58–172)	15.6 #	111 (60–194)	17.6

# Proportional contributions of each source to total, beverage-, and food-derived energy differed significantly across groups (*p* < 0.05).

**Table 6:** Energy intake (kcal/day) and source-specific contributions (%), stratified by whether total water intake met the adequate intake recommended by the Chinese Nutrition Society.

Variables	Group 1 (*n* = 131, 19.4%)		Group 2 (*n* = 545, 80.6%)	
	Median (P25–P75)	%	Median (P25–P75)	%
Total energy intake	1246 (1050–1620) *	–	919 (721–1164)	–
Energy from beverages	245 (153–400) *	23.2	173 (81–342)	22.7
Plain water	0 (0–0)	0	0 (0–0)	0
MMDs	209 (119–381) *	84.7	140 (54–273)	82.8
FVDs	0 (0–8) *	3.1	0 (0–0)	2.9
SSBs	0 (0–10) *	10.1	0 (0–3)	9.4
BPDs	0 (0–1)	1.8 #	0 (0–0)	4.7
Hot beverages	0 (0–0)	0.3	0 (0–0)	0.2
Energy from foods	967 (793–1192) *	76.8	701 (529–878)	77.3
Staple foods	364 (259–467) *	37.7 #	279 (197–387)	41.5
Dishes	360 (269–483) *	38.8	253 (197–325)	38.7
Porridge	15 (2–99) *	6.3 #	5 (1–31)	3.4
Soup	0 (0–0)	0	0 (0–0)	0
Snacks	147 (83–246) *	17.2	97 (56–171)	16.4

Group 1: children whose daily TWI met the AI recommended by the CNS; Group 2: children whose daily TWI did not meet the AI. * Group differences in energy intake from specific sources were statistically significant (*p* < 0.05); # proportional contributions of each source to total, beverage-, and food-derived energy differed significantly across groups (*p* < 0.05).

**Table 7:** Partial correlations between water and energy intake from various sources, adjusted for age in months, sex, height, and weight.

Variables	Total Water Intake	Total Fluid Intake	Water from Beverages	Water from Foods	Total Energy Intake	Energy from Beverages	Energy from Foods
Total water intake	1.000						
Total fluid intake	0.817 ***	1.000					
Water from beverages	0.824 ***	0.990 ***	1.000				
Plain water	0.732 ***	0.888 ***	0.903 ***	0.123 **	0.086 *	0.072	0.057
MMDs	0.367 ***	0.437 ***	0.443 ***	0.087 *	0.274 ***	0.328 ***	0.103 **
FVDs	0.163 ***	0.196 ***	0.181 ***	0.053	0.130 ***	0.126 **	0.073
SSDs	0.153 ***	0.138 ***	0.132 ***	0.099 *	0.195 ***	0.225 ***	0.081 *
BPDs	0.075	0.084 *	0.082 *	0.027	0.059	0.022	0.060
Hot beverages	0.055	0.033	0.028	0.062	0.033	0.006	0.039
Water from foods	0.691 ***	0.161 ***	0.161 ***	1.000			
Staple foods	0.247 ***	–0.022	–0.018	0.453 ***	0.401 ***	0.007	0.517 ***
Dishes	0.539 ***	0.178 ***	0.175 ***	0.715 ***	0.531 ***	0.091 *	0.623 ***
Porridge	0.306 ***	0.031	0.038	0.484 ***	0.035	–0.143 ***	0.156 ***
Soup	0.404 ***	0.159 ***	0.150 ***	0.512 ***	0.250 ***	0.118 **	0.235 ***
Snacks	0.363 ***	0.107 **	0.100 **	0.505 ***	0.415 ***	0.037	0.513 ***
Total energy intake	0.483 ***	0.276 ***	0.223 ***	0.557 ***	1.000	0.644 ***	0.806 ***
Energy from beverages	0.178 ***	0.313 ***	0.234 ***	0.012	0.644 ***	1.000	0.067
MMDs	0.142 ***	0.287 ***	0.206 ***	–0.016	0.572 ***	0.880 ***	0.066
FVDs	0.107 **	0.158 ***	0.134 ***	0.016	0.117 **	0.145 ***	0.041
SSBs	0.085 *	0.086 *	0.078 *	0.048	0.255 ***	0.427 ***	0.003
BPDs	0.075	0.084 *	0.082 *	0.027	0.059	0.022	0.060
Hot beverages	0.055	0.033	0.028	0.062	0.033	0.003	0.039
Energy from foods	0.492 ***	0.118 **	0.11 **	0.717 ***	0.806 ***	0.067	1.000
Staple foods	0.225 ***	–0.012	–0.010	0.405 ***	0.549 ***	–0.008	0.723 ***
Dishes	0.485 ***	0.191 ***	0.179 ***	0.617 ***	0.596 ***	0.123 **	0.682 ***
Porridge	0.321 ***	0.076 *	0.081 *	0.456 ***	0.146 ***	–0.082 *	0.254 ***
Snacks	0.227 ***	0.061	0.049	0.333 ***	0.545 ***	0.085 *	0.645 ***

* *p* < 0.05; ** *p* < 0.01; and *** *p* < 0.001.

## Data Availability

The datasets generated and/or analyzed during this study are available from the corresponding author on reasonable request. Public access is not provided due to privacy and ethical considerations.

## Supplementary Materials

The following supporting information can be downloaded at: https://www.mdpi.com/article/10.3390/nu17162645/s1, Figure S1: The characteristics of participants; Table S2: Water intake (mL/day) and source-specific contributions (%) among Chinese preschool children in 2018, stratified first by age group (months), then by sex; Table S3: Energy intake (kcal/day) and source-specific contributions (%) among Chinese preschool children in 2018, stratified first by age group (months), then by sex.

## Abbreviations

TWI	Total water intake
CNS	Chinese Nutrition Society
DSIYC	Dietary intake survey of infants and young children
TEI	Total energy intake
AI	Adequate intake
TFI	Total fluid intake
MMDs	Milk and milk derivatives
FVDs	Fruit and vegetables drinks
SSBs	Sugar-sweetened beverages
BPDs	botanical protein drinks
