# Supplementary material for: Total Water and Energy Intake Among Preschool Children in China: A Cross-Sectional Analysis Based on National Survey Data
Source: Nutrients. 2025 Aug 15;17(16):2645. doi: 10.3390/nu17162645 (PMC12389339; doi:10.3390/nu17162645)
Supplement: Supplementary file 1 [file nutrients-17-02645-s001.zip › nutrients-3803306-supplementary.pdf]

Table S1. The characteristics of participants

Variables	Total	37–48 months	49–60 months	61–72 months
**N**	676 (100)	224 (33.1)	226 (33.4)	226 (33.4)
**Age (month)**	54.6 ± 10.2 *	43.1 ± 3.5	54.0 ± 3.5	66.6 ± 3.7
**Height (cm)**	106.8 ± 7.9 *	100.4 ± 5.0	106.4 ± 5.3	113.6 ± 6.8
**Weight (kg)**	18.1 ± 3.5 *	15.8 ± 2.1	18.0 ± 2.8	20.7 ± 3.6

Values are shown as the n (%) or the mean ± standard deviation; * Group differences in age, height, and weight were statistically significant ($p < 0.05$).

Table S2. Water intake (mL/day) and source-specific contributions (%) among Chinese preschool children in 2018, stratified first by age group (months), then by sex.

Variables	37–48 months (*n* = 224)				49–60 months (*n* = 226)				61–72 months (*n* = 226)			
	Boy (*n* = 114)		Girl (*n* = 110)		Boy (n = 105)		Girl (*n* = 121)		Boy (*n* = 121)		Girl (*n* = 105)	
	Median (P25–P75)	%	Median (P25–P75)	%	Median (P25–P75)	%	Median (P25–P75)	%	Median (P25–P75)	%	Median (P25–P75)	%
**Total water intake**	1312 (1000–1560) *	–	1087 (926–1377)	–	1280 (1053–1523)	–	1201 (974–1497)	–	1220 (950–1575)	–	1254 (992–1565)	–
**Total fluid intake**	785 (600–1001) *	–	623 (494–853)	–	735 (534–955)	–	675 (543–967)	–	713 (509–991)	–	650 (511–869)	–
**Water from beverages**	751 (575–966) *	59.5	602 (473–830)	56.4	674 (500–894)	53.6	618 (502–890)	54.6	689 (495–942)	56.1	624 (496–829)	54.0
**Plain water**	500 (350–800) *	71.1	450 (300–600)	73.7	500 (315–775)	78.0	500 (350–750)	76.7	500 (325–750)	73.0	500 (300–600)	74.1
**MMDs**	179 (104–266) *	26.1	150 (63–224)	22.6	103 (4–204)	17.6	114 (8–209)	18.9	130 (44–221)	20.0	135 (67–196)	20.1
**FVDs**	0 (0–0)	0.3	0 (0–0)	0.3	0 (0–0)	0.6	0 (0–0)	0.7	0 (0–0)	1.0	0 (0–0)	1.0
**SSBs**	0 (0–0)	1.3	0 (0–14)	2.1	0 (0–24)	2.2	0 (0–24)	2.7	0 (0–46)	4.1	0 (0–26)	2.9
**BPDs**	0 (0–0)	1.2	0 (0–0)	1.2	0 (0–16)	1.6	0 (0–0)	1.1	0 (0–8)	1.9	0 (0–23)	2.0
**Hot beverages**	0 (0–0)	0.0	0 (0–0)	0.0	0 (0–0)	0.0	0 (0–0)	0.0	0 (0–0)	0.0	0 (0–0)	0.0
**Water from foods**	469 (361–652)	40.5	459 (358–596)	43.6	571 (455–714)	46.4	524 (416–689)	45.4	503 (378–659)	43.9	569 (390–763)	46.0
**Staple foods**	130 (92–181)	29.8	118 (93–169)	29.3	139 (105–205)	27.9	134 (99–172)	26.7	152 (120–199)	33.1	147 (109–206)	29.7
**Dishes**	141 (84–200)	29.0	123 (78–176)	27.0	135 (89–206)	25.9	116 (79–200)	24.9	134 (85–213)	27.8	148 (101–207)	28.5
**Porridge**	50 (18–111)	15.3	41 (18–118)	15.7	83 (24–184)	20.6	98 (48–177)	22.3	38 (11–92)	12.3	28 (0–111)	12.3
**Soup**	50 (20–108)	13.7	50 (25–94)	13.9	65 (25–135)	13.8	70 (30–134)	15.3	75 (25–126)	15.1	75 (25–142)	16.6
**Snacks**	51 (28–83)	12.1	51 (31–105)	14.1	58 (24–101)	11.8	58 (26–85)	10.8	50 (25–88)	11.6	60 (32–110)	13.0

MMDs: milk and milk derivatives; FVDs: fruit and vegetables drinks; SSBs: sugar sweetened beverages; BPDs: botanical protein drinks;
* Group differences in water intake from specific sources were statistically significant ($p < 0.05$).

Table S3. Energy intake (kcal/day) and source-specific contributions (%) among Chinese preschool children in 2018, stratified first by age group (months), then by sex.

Variables	37–48 months (n = 224)				49–60 months (n = 226)				61–72 months (n = 226)			
	Boy（n = 114）		Girl（n = 110）		Boy（n = 105）		Girl（n = 121）		Boy（n = 121）		Girl（n = 105）	
	**Median (P25–P75)**	**%**	**Median (P25–P75)**	**%**	**Median (P25–P75)**	**%**	**Median (P25–P75)**	**%**	**Median (P25–P75)**	**%**	**Median (P25–P75)**	**%**
**Total energy intake**	957 (719–1219)	–	863 (692–1107)	–	1000 (775–1231)	–	986 (773–1241)	–	1032 (811–1257)	–	1071 (833–1422)	–
**Energy from beverages**	242 (130–367) *	26.4 #	159 (91–326)	21.9	176 (95–333)	21.3	196 (100–377)	23.5	184 (78–359)	21.2	213 (78–413)	22.3
**Plain water**	0 (0–0)	0.0	0 (0–0)	0.0	0 (0–0)	0.0	0 (0–0)	0.0	0 (0–0)	0.0	0 (0–0)	0.0
**MMDs**	235 (123–360) *	91.2	137 (74–305)	88.0	144 (69–250)	81.1	152 (75–295)	84.6	126 (46–233) *	73.8	166 (54–347)	80.5
**FVDs**	0 (0–0)	2.1	0 (0–0)	1.4	0 (0–0)	3.8	0 (0–0)	3.1	0 (0–0)	3.3	0 (0–0)	4.1
**SSBs**	0 (0–0)	3.4	0 (0–0)	6.9	0 (0–5)	10.5	0 (0–3)	8.7	0 (0–21)	16.8	0 (0–6)	10.8
**BPDs**	0 (0–0)	3.3	0 (0–0)	3.5	0 (0–5	3.9	0 (0–0)	3.5	0 (0–3)	6.1	0 (0–8)	4.3
**Hot beverages**	0 (0–0)	0.0	0 (0–0)	0.3	0 (0–0)	0.7	0 (0–0)	0.0	0 (0–0)	0.0	0 (0–0)	0.4
**Energy from foods**	661 (493–895)	76.3 #	685 (508–883)	78.1	748 (598–970)	78.7	729 (547–912)	76.5	794 (610–998)	78.8	847 (660–1033)	77.7
**Staple foods**	256 (183–401)	40.4	277 (186–353)	40.7	314 (219–417) *	40.7	282 (202–349)	38.7	327 (235–482)	43.1	317 (244–469)	41.0
**Dishes**	250 (185–323)	39.0	234 (172–310)	36.9	280 (229–360)	39.8	274 (208–363)	40.0	283 (205–390)	39.0	284 (226–387)	37.5
**Porridge**	5 (1–27)	3.9	5 (1–33)	4.4	12 (3–39)	4.1	12 (4–53)	4.5	3 (1–32)	3.1	2 (0–38)	3.6
**Soup**	0 (0–0)	0.0	0 (0–0)	0.0	0 (0–0)	0.0	0 (0–0)	0.0	0 (0–0)	0.0	0 (0–0)	0.0
**Snacks**	101 (66–161)	16.7	110 (60–182)	18.1	97 (57–161)	15.4	108 (55–185)	16.9	98 (54–185)	14.8 #	118 (67–214)	17.9

MMDs: milk and milk derivatives; FVDs: fruit and vegetables drinks; SSBs: sugar sweetened beverages; BPDs: botanical protein drinks; * Group differences in energy intake from specific sources were statistically significant ($p < 0.05$); # Proportional contributions of each source to total, beverage-, and food-derived energy differed significantly across groups ($p < 0.05$)
